# Long non‐coding RNA as a potential diagnostic and prognostic biomarker in melanoma: A systematic review and meta‐analysis

**DOI:** 10.1111/jcmm.18109

**Published:** 2024-01-09

**Authors:** Mahdi Masrour, Shaghayegh Khanmohammadi, Parisa Fallahtafti, Seyedeh Melika Hashemi, Nima Rezaei

**Affiliations:** ^1^ School of Medicine Tehran University of Medical Sciences Tehran Iran; ^2^ Research Center for Immunodeficiencies, Pediatrics Center of Excellence, Children's Medical Center Tehran University of Medical Sciences Tehran Iran; ^3^ Non‐Communicable Diseases Research Center, Endocrinology and Metabolism Population Sciences Institute Tehran University of Medical Sciences Tehran Iran; ^4^ Tehran Heart Center, Cardiovascular Diseases Research Institute Tehran University of Medical Sciences Tehran Iran; ^5^ Department of Immunology, School of Medicine Tehran University of Medical Sciences Tehran Iran

**Keywords:** biomarker, diagnosis, diagnostic value, lncRNA, long noncoding RNA, melanoma, prognosis, prognostic value

## Abstract

Recently, long noncoding RNAs (lncRNAs) have been applied as biomarkers for melanoma patients. In this systematic review and meta‐analysis, we investigated the diagnostic and prognostic value of lncRNAs. We used the keywords ‘lncRNA’ and ‘melanoma’ to search databases for studies published before June 14th, 2023. The specificity, sensitivity and AUC were utilized to assess diagnostic accuracy and the prognostic value was assessed using overall survival, progression‐free survival and disease‐free survival hazard ratios. After screening 1191 articles, we included seven studies in the diagnostic evaluation section and 17 studies in the prognosis evaluation section. The Reitsma bivariate model estimated a cumulative sensitivity of 0.724 (95% CI: 0.659–0.781, *p* < 0.001) and specificity of 0.812 (95% CI: 0.752–0.859, *p* < 0.001). The pooled AUC was 0.780 (95% CI: 0.749–0.811, *p* < 0.0001). The HR for overall survival was 2.723 (95% CI: 2.259–3.283, *p* < 0.0001). Two studies reported an HR for overall survival less than one, with an HR of 0.348 (95% CI: 0.200–0.607, *p* < 0.0002). The HR for progression‐free survival was 2.913 (95% CI: 2.050–4.138, *p* < 0.0001). Four studies reported an HR less than one, with an HR of 0.457 (95% CI: 0.256–0.817). The HR for disease‐free survival was 2.760 (95% CI: 2.009–3.792, *p* < 0.0001). In conclusion, the expression of lncRNAs in melanoma patients affects survival and prognosis. LncRNAs can also be employed as diagnostic biomarkers.

## 1 INTRODUCTION

Melanoma is one of the most prevalent cancers worldwide, with an estimated prevalence of 3.7 cases per 100,000 persons in 2020, according to global statistics (GLOBOCAN).^1^ Melanoma, the most severe type of cutaneous malignancy, is the leading cause of mortality from skin cancer.^2^ Despite recent advances in melanoma treatment options, such as immunotherapy and targeted molecular therapy, its high mortality rate remains challenging.^3^ There are varied uncertainties around diagnosis, clinical decision‐making and treatment of melanoma. However, significant progress in genetic, epigenetic and transcriptomic fields has shown great promise for developing potential biomarkers for diagnosing and determining patients' prognoses.^4^

Melanoma occurs due to abnormalities in multiple genes and signalling pathways controlling cell proliferation and function, which itself arises from the alternation in either gene sequence or expression.^5^ Besides the genetic predisposition, so far, extensive attention is being paid to epigenetic events involved in the initiation or progression of melanoma.^5^ Noncoding RNAs (ncRNAs) are a new class of regulatory molecules associated with diseased conditions like different types of cancers. Long noncoding RNAs (lncRNAs) are noncoding transcripts longer than 200 nucleotides involved in much of the gene life cycle, including transcriptional, posttranscriptional and epigenetic mechanisms of gene regulation.^6^

Various types of RNAs have recently been applied as a biomarker for disease detection,^7^, ^8^ but still, there are scarce specified diagnostic panels. Identifying shared lncRNA dysregulation may lend insight into the early patient's diagnosis and prognosis and find potentially novel targets for treatment. With the advancements in sequencing technologies, many studies have shown over‐ or under‐expression of specific types of lncRNAs, such as NKILA, PVT1, FDG5‐AS1 and HOXA6, in patients with melanoma.^9^, ^10^, ^11^ However, indicating its diagnostic accuracy is important for the clinical application of these biomarkers. Also, the prognostic accuracy of these biomarkers must be identified for future clinical applications.^10^

Reviews of the literature describe the disruption of lncRNA expression within cancer types, but lncRNA use as a diagnostic and prognostic biomarker has not been systematically reviewed across melanoma patients. To the best of our knowledge, the present study is the first systematic review and meta‐analysis concerned with this issue. We recorded lncRNA‐related diagnostic and prognostic values from articles that extracted lncRNAs from human tissue specimens retrieved from melanoma patients.

## 2 METHODS

We conducted a systematic review and meta‐analysis in accordance with the PRISMA guidelines.^12^ Our systematic review and meta‐analysis protocol has been registered at PROSPERO with the registration number CRD42023441549.

### 2.1 Literature search

An in‐depth search was performed until 14th June 2023, in PubMed, Web of Science (ISI), Scopus and Embase to identify English publications without any limitations on publication year. Databases were searched by the following medical subject headings (MeSH) terms and free keywords: ‘long non‐coding RNA’ and ‘melanoma’ and their expansions. Table S1 provides the search query.

### 2.2 Selection criteria

This study incorporated original research that had previously been reviewed by peers and presented the sensitivity, specificity or area under the curve (AUC) values of lncRNAs in the diagnosis of melanoma, as well as their association with prognosis in terms of overall survival (OS), progression‐free survival (PFS), disease‐free survival (DFS), recurrence‐free survival (RFS) and event‐free survival (EFS). While the diagnostic part of our research consisted of novel case–control human studies, the prognostic part employed cohort studies. The research studies were carried out in either a prospective or a retrospective manner, and they used samples acquired from patients who had been pathologically diagnosed with melanoma as well as those who were healthy as controls. In diagnostic accuracy studies, the comparison of lncRNA to an adequate reference control should have been performed regardless of the test assay time in order to evaluate sensitivity, specificity and AUC. There were no limitations placed on eligibility based on the healthcare settings in which the research was carried out, nor were there any limitations placed on eligibility based on the total number of participants in the studies that were included. Non‐English studies, studies on datasets or animal models, letters, comments, reviews, editorials, conference abstracts, case reports and case series were considered ineligible and were therefore excluded from the analysis.

Following the removal of any duplicates, SK and PF went through the remaining identified papers and evaluated their eligibility based on the inclusion and exclusion criteria that had been previously outlined. After compiling a list of studies that satisfied the eligibility requirements, both authors proceeded to independently conduct a comprehensive review of the full texts of the studies. During the review process, any conflicts that arose were effectively resolved through the formation of a consensus.

### 2.3 Data extraction

Two reviewers (SMH and PF) independently extracted data from the included studies in a dedicated electronic spreadsheet. The following data were extracted from each when available: author, publication year, specimen type, sample size, control population, lncRNA name, change in levels of lncRNA in patients compared to the control group, diagnostic or prognostic performance measures, including sensitivity, specificity, AUC with corresponding 95% confidence interval (CI) and *p*‐value, as well as mean, median and hazard ratio (HR) for survival outcomes with corresponding 95% CI and *p*‐Value. Discrepancies were resolved through discussion and consensus.

### 2.4 Quality assessment

The quality of the included studies was assessed using an appropriate tool, the Newcastle‐Ottawa Scale (NOS), for cohort and case–control studies.^13^ Two reviewers (PF and SMH) independently assessed the quality of each study based on predefined criteria. Any discrepancies in the quality assessment were resolved through discussion or consultation with a third reviewer. Selection, comparability and outcome are the three main categories of bias in NOS. Scores of 7 and above, 2–6, 1 and below were considered ‘good’, ‘fair’ and ‘poor’, respectively.

### 2.5 Statistical analysis

We used the bivariate random effect model that was developed by Reitsma et al. (2005) in order to compile the research that provides diagnostic specificity and sensitivity.^14^ A bivariate model uses logit transformation to aggregate test sensitivity and specificity across studies. This is done by taking into account the interdependency of the two variables. This model also determines the summary receiver operating characteristic (sROC) curve and the AUC, both representing the accuracy of the diagnosis. For the studies that reported AUCs, the inverse variance method was used to meta‐analyse AUC values. Because it was expected that there would be heterogeneity across the research included, the random effects model was used.

We employed the inverse variance method with logarithmic HR values to conduct a meta‐analysis of prognostic values, which were reported as HRs. The random effects model was used to account for the observed heterogeneity in the reported values. Because HR less than one indicates that the explored variable (lncRNAs) has a cancer‐protective function and HR more than one shows that the lncRNA has a cancer‐promoting function, these two groups were split as distinct categories independent of the regulation of the studied lncRNA.

The standard error of the AUC and HRs for meta‐analysis was calculated using either the 95% CI or the AUC value and sample size if the CI was not available. The study employed *I* ^2^ and the DerSimonian‐Laird estimator of tau2 statistics to assess research heterogeneity. A subgroup analysis was conducted based on the sample type obtained to further explore the heterogeneity. The statistical analysis and visualizations were conducted using R version 4.2.2. Statistical significance was determined by an *I* ^2^ value exceeding 50% and a *p*‐value below 0.05.

## 3 RESULTS

### 3.1 Basic characteristics

**FIGURE 1:**
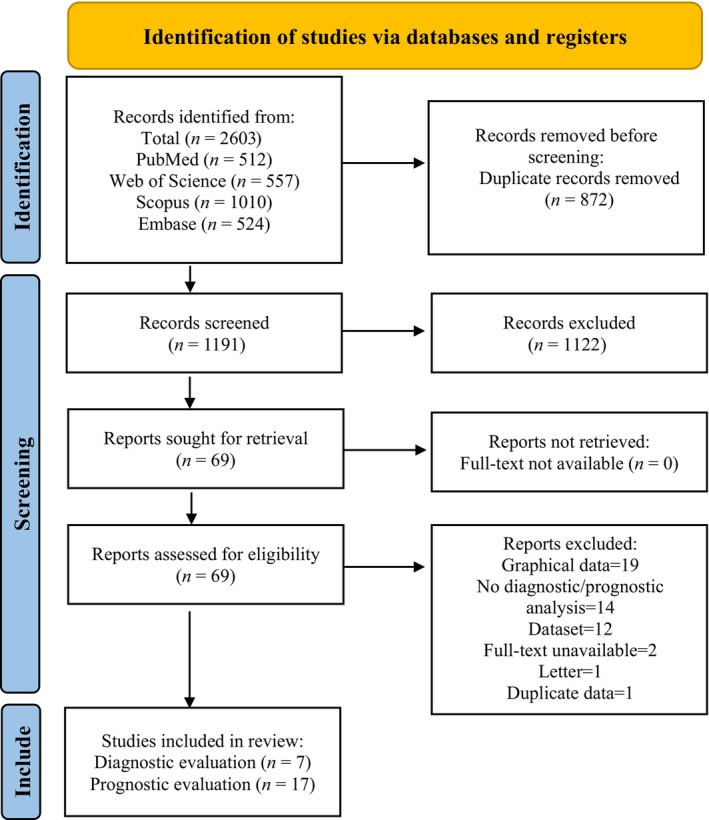
The PRISMA flowchart.

After performing the initial database search, a total of 2603 titles were obtained. After eliminating duplicate articles, a total of 1191 articles were screened. After a comprehensive review of titles and abstracts, 1122 articles were excluded, and a total of 69 articles were considered appropriate for full‐text review. A total of seven studies met the inclusion criteria for the diagnostic accuracy section, while 17 studies met the inclusion criteria for the prognosis section. The excluded studies are listed in Table S2. The PRISMA flowchart, shown in Figure 1, outlines the process of selecting and excluding studies.

Table 1 provides a concise overview of the fundamental characteristics of the studies that were included. Overall, 647 melanoma patients and 721 controls were studied in the included studies for the diagnostic values, and 1453 melanoma patients were studied for the prognostic values. The papers included in the diagnosis section were published from 2016 to 2022, while those included in the prognosis section were published from 2014 to 2023. One study reported the diagnostic and prognostic value of more than one lncRNA.^10^ The meta‐analysis of diagnosis accuracy comprised a total of 1579 melanoma samples and 805 healthy samples obtained from China and Poland. The prognosis section analysed a total of 1563 melanoma samples from China and Poland to assess OS. Additionally, 580 cases from Poland were examined to evaluate PFS, and 576 cases from China were used to assess DFS. The meta‐analysis of diagnostic evaluations consisted of 24 evaluations, which involved two types of specimens: four tumour tissue samples and 20 blood samples. The prognostic evaluations encompassed two types of specimens: tumour tissue samples and blood samples.

**TABLE 1:** Basic characteristics of the included studies.

Prognosis section														
ID	Author, year	Country	Specimen	Control type	Case N.	Control N.	lncRNA	Up/downregulation	Overall survival [HR] (95% CI)	*p*‐Value	Progression‐free survival [HR] (95% CI)	*p*‐Value	Disease‐free survival [HR] (95% CI)	*p*‐Value
1	An, 2019	China	Tissue	Adjacent normal tissue	30	30	LncRNA H19	Up	1.4	0.012				
2	Bai, 2021	China	Tissue	Adjacent normal tissue	137	137	NR2F1‐AS1	Up	2.986 (1.348–4.832)	0.015			3.013 (1.448–5.231)	0.004
3	Bao, 2014	China	Tissue	Melanocytic nevus	103	12	BANCR	Up	Median: ‐high: 13.055 months ‐low: 55.021 months					
4	Gao, 2020	China	Tissue	Adjacent normal tissue	188	188	FDG5‐AS1	Up	2.985 (1.218–4.652)	0.009			2.814 (1.382–4.765)	0.003
5	Ji, 2023	China	Tissue	Adjacent normal tissue	30	30	LINC00467	Up	0.54	0.22			0.31	0.42
6	Kolenda, 2019	Poland	Serum	Nonmelanoma subjects	58	15	antiPeg11	Down	3.31 (1.64–9.16)	0.046	3.11 (1.65–8.86)	0.0486		
							HOTAIR	Down	1.07 (0.32–3.62)	0.9114	0.54 (0.13–2.17)	0.54		
							IGF2AS	Up	3.52 (1.9–11.06)	0.049	3.37 (1.93–8.9)	0.046		
							MEG3	Down	3.02 (1.63–10.73)	0.0393	3.1 (2.01–7.09)	0.0499		
							Nespas	Down	1.55 (0.55–4.39)	0.4067	0.83 (0.29–2.41)	0.7308		
							PCGEM1	Down	1.85 (0.53–6.4)	0.3339	1.36 (0.4–4.56)	0.6222		
							PSFinhibitingRNA	Down	1.92 (0.58–6.38)	0.2861	1.83 (0.53–6.29)	0.3375		
							Sox2ot	Down	4.15 (1.93–12.78)	0.019	3.86 (1.64–10.75)	0.045		
							SNHG1	Down	0.42 (0.19–0.92)	0.0314	0.48 (0.17–1.38)	0.1722		
							Zeb2NAT	Down	0.29 (0.13–0.62)	0.0015	0.24 (0.09–0.63)	0.0039		
7	Liu C, 2022	China	Tissue	Adjacent normal tissue	65	65	LNCOC1	Up	1.862	0.008				
8	Liu, 2016	China	Serum	Nonmelanoma subjects	70	79	SPRY4‐IT1	Up	2.931 (1.103–7.79)	0.031				
9	Luan, 2019	China	Tissue	Adjacent normal tissue	30	30	OIP‐AS1	Up	3.135 (1.167–8.426)	0.023				
10	Huang, 2019	China	Tissue	Adjacent normal tissue	104	104	DSCAM‐AS1	Up	3.016 (1.126–4.219)	0.009				
11	Gao H, 2019	China	Tissue	Adjacent normal tissue	148	148	SNHG17	Up	2.856 (1.138–4.327)	0.007				
12	Xu, 2020	China	Tissue	Adjacent normal tissue	60	60	LUADT1	Up	2.078	0.017				
13	Xu, 2020	China	Tissue	Nonmelanoma subjects	NA	NA	FOXC2‐AS1	Up	1.933	0.0487				
14	Wei, 2017	China	Tissue	Adjacent normal tissue	88	88	ZFAS1	Up	2.735 (1.245–4.969)	0.001			2.602 (1.289–4.439)	0.001
15	Wang, 2019	China	Tissue	Benign nevi	55	30	TUG1	Up	2.44 (1.57–3.78)	0.048				
16	Wang, 2022	China	Tissue	Adjacent normal tissue	163	163	LINC00173	Up	2.768 (1.157–4.456)	0.014			2.629 (1.158–4.385)	0.017
17	Ren, 2019	China	Tissue	Normal tissue	124	40	FOXD2‐AS1	Up	1	0.76			1	0.96

Diagnosis section												
ID	Author, year	Country	Specimen	Control type	Case N.	Control N.	lncRNA	Up/downregulation	Sensitivity	Specificity	AUC (95% CI)	*p*‐Value
1	Bai, 2021	China	Tissue	Adjacent normal tissue	137	137	NR2F1‐AS1	Up	68.56	84.37	0.7365 (0.6777–0.7953)	
2	Bian, 2017	China	Tissue	Adjacent normal tissue	92	92	NKILA	Down			0.875	<0.0001
3	Chen X, 2017	China	Serum	Nonmelanoma subjects	51	47	PVT1	Up	94.12	85.11	0.9387 (0.8899–0.9874)	
				Nonmelanoma subjects (vs. stage I)	51	47	PVT1	Up	87.5	85.11	0.8684 (0.7611–0.9756)	
4	Kolenda, 2019	Poland	Serum	Nonmelanoma subjects	58	15	CAR Intragenic 10	Up	68.09	90.91	0.807	<0.0001
							NDM29	Down	61.54	90.91	0.792	<0.0001
							H19 antisense	Down	51.79	100	0.791	<0.0001
							HOXA6as	Down	100	61.54	0.787	0.0012
							NRON	Down	76.79	76.92	0.78	0.0001
							Zfhx2as	Down	100	50	0.769	0.0004
							IPW	Down	53.66	100	0.767	0.0008
							BC200	Down	64.15	84.62	0.763	0.0002
							UM9‐5	Up	72.73	80	0.759	<0.0001
							WT1‐AS	Down	95.45	61.54	0.753	0.0038
							Kcnq1ot1	Up	72.73	77.78	0.735	0.0027
							Tsix	Up	56.25	100	0.731	0.0003
							IGF2as (family)	Up	51.79	91.67	0.707	0.0141
							SNHG3	Down	83.64	50	0.703	0.0219
							SNHG1	Down	76.79	61.54	0.696	0.0164
							E2F2 antisense	Down	65.31	72.73	0.692	0.0186
							HOTAIR	Down	58.14	80	0.681	0.0221
5	Liu, 2016	China	Serum	Nonmelanoma subjects	70	79	SPRY4‐IT1	Up	72.2	82.4	0.813	
6	Xiao, 2019	China	Tissue	Normal tissue	40	16	LINC0638	Up			0.8734 (0.7699–0.97)	<0.0001
				Benign skin lesion	40	23	LINC0638	Up			0.837 (0.738–0.9359)	<0.0001
			Plasma	Normal tissue	40	16	LINC0638	Up			0.8391 (0.7287–0.9494)	<0.0001
				Benign skin lesion	40	23	LINC0638	Up			0.8136 (0.7097–0.9174)	<0.0001
7	Wang, 2022	China	Tissue	Adjacent normal tissue	163	163	LINC00173	Up	76	85	0.7695 (0.7172–0.8217)	<0.001
				I/II tumour stage melanoma tissue	53	110	LINC00173	Up			0.7451 (0.6512–0.8389)	<0.001

Among the 24 diagnostic evaluations conducted in the studies analysed, there were 23 distinct lncRNAs. Eleven diagnostic evaluations reported upregulation of lncRNAs, while 13 evaluations reported their downregulation. A total of 21 diagnostic evaluations have reported the sensitivity and specificity metrics for diagnosing melanoma. All of these 21 evaluations were conducted on distinct types of lncRNA.

The meta‐analysis of prognostic evaluations consisted of 19 evaluations. Nineteen distinct lncRNAs were assessed in the meta‐analysed studies. In 10 prognostic evaluations, upregulation of lncRNAs was observed, while downregulation was observed in nine evaluations.

### 3.2 Quality assessment

The studies included in the analysis were assessed for quality using the NOS by independent investigators (Table 2). Fourteen studies received a ‘good’ score, seven studies received a ‘fair’ score, and no studies received a ‘poor’ score, indicating a low risk of bias for included studies.

**TABLE 2:** The Newcastle‐Ottawa Scale quality assessment.

ID	Author, year	Selection				Comparability	Exposure			Overall score
		Case definition	Representativeness	Selection of controls	Definition of controls		Ascertainment of exposure	The same method of ascertainment	Nonresponse rate	
1	An, 2019	*			*	**		*	*	6
2	Bai, 2021	*	*		*	**		*	*	7
3	Bao, 2014	*	*		*	**	*	*	*	8
4	Bian, 2017	*	*		*	**	*	*	*	8
5	Chen X, 2017	*	*	*	*	**	*	*	*	9
6	Gao H, 2019	*	*			**	*	*	*	7
7	Gao, 2020	*	*			**	*	*	*	7
8	Huang, 2019	*	*			**	*	*	*	7
9	Ji, 2023	*				**	*	*	*	6
10	Kolenda, 2019	*	*	*	*	**	*	*	*	9
11	Liu C, 2022	*	*			**	*	*	*	7
12	Liu, 2016	*	*	*	*	**	*	*	*	9
13	Luan, 2019	*				**	*	*	*	6
14	Xu, 2020	*			*	**		*	*	6
15	Xu, 2020					**		*		3
16	Wei, 2017	*	*	*	*	**	*	*	*	9
17	Wang, 2019	*				**	*	*		5
18	Wang, 2022	*	*	*	*	**	*	*	*	9
19	Ren, 2019	*			*	**		*	*	6
20	Xiao, 2019	*	*	*	*	**	*	*	*	9

* indicate whether the study in a row received a score for each of the columns.

### 3.3 Meta‐analysis of diagnostic value of lncRNAs in melanoma patients

**FIGURE 2:**
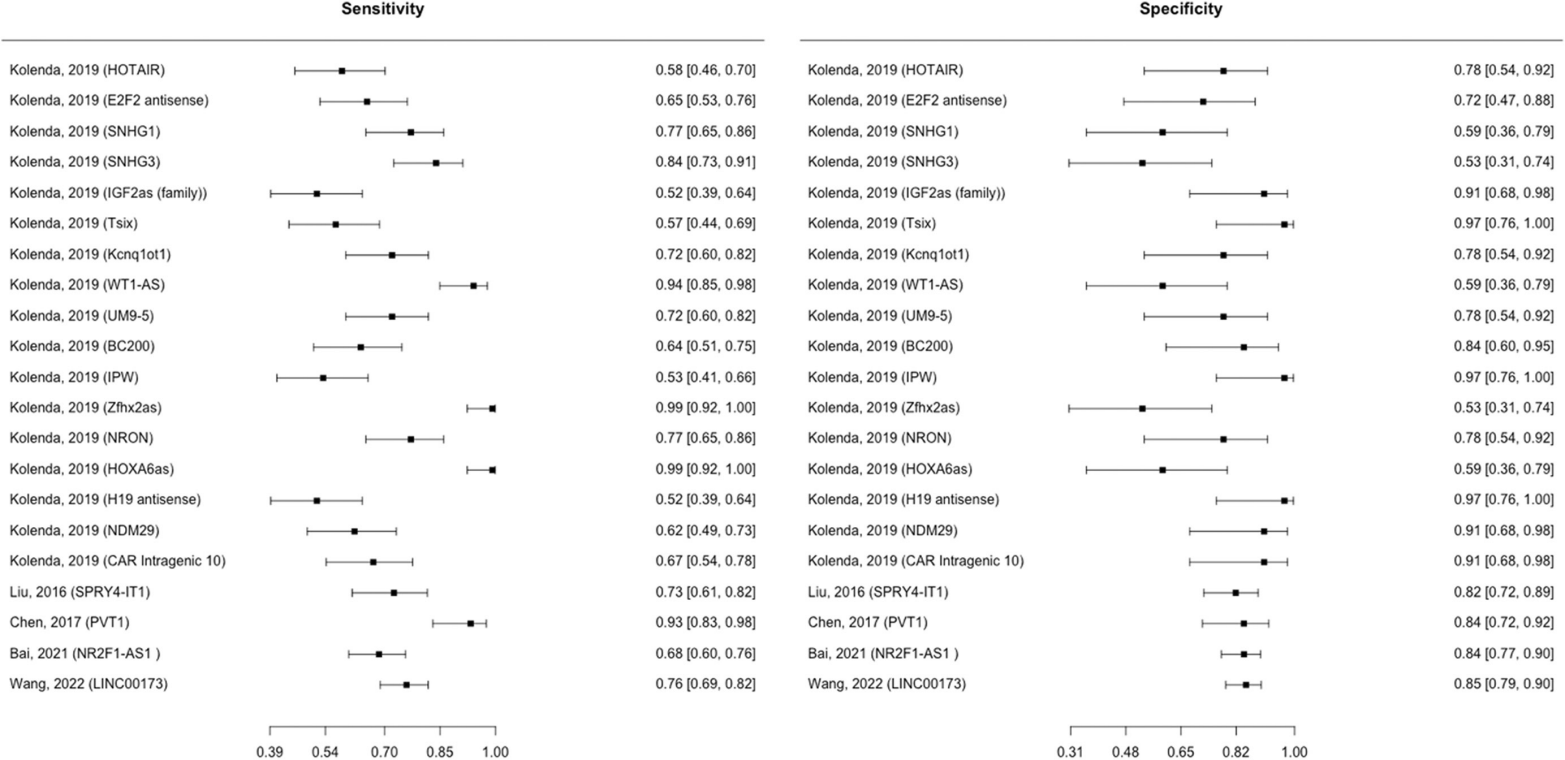
Diagnostic accuracy of lncRNAs.

**FIGURE 3:**
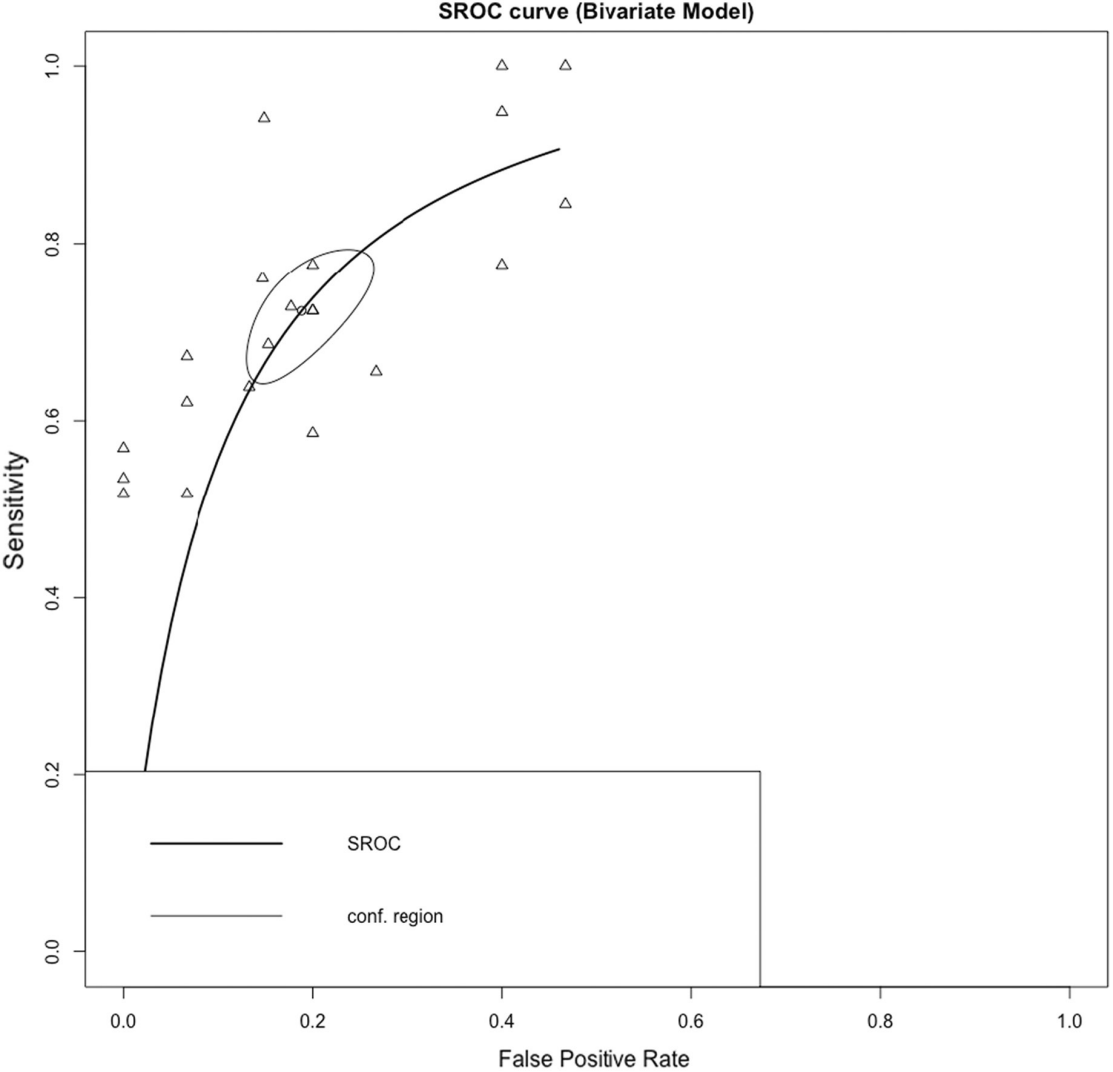
The summary receiver operating characteristics (sROC) curve was plotted using the sensitivities and false positive rates of included studies.

The Reitsma bivariate model estimated a cumulative sensitivity of 0.724 (95% CI: 0.659–0.781, *p* < 0.001) and a pooled specificity of 0.812 (95% CI: 0.752–0.859, *p* < 0.001) for lncRNAs in diagnosing melanoma involving 1407 melanoma cases and 681 controls (Figure 2). The estimated *I* ^2^ value using the Holling sample size unadjusted approach was 14.9%–32.5%. The test for equality of sensitivities among the studies had a *p*‐value of <2e‐16, and the test for equality of specificities had a *p*‐value of 0.000599. The sROC curve was generated, and the overall pooled AUC for all specimen types was determined to be 0.837 (Figure 3). For lncRNAs in blood specimens (*n* = 19), involving 1107 melanoma cases and 381 controls, the Reitsma bivariate model indicated a cumulative sensitivity of 0.720 (95% CI: 0.648–0.783, *p* < 0.001) and a pooled specificity of 0.794 (95% CI: 0.724–0.851, *p* < 0.001) in detecting melanoma. Holling sample size unadjusted *I* ^2^ was 18.7–33%. The *p*‐value for the test for equality of sensitivities across studies was <2e‐16, while the *p*‐value for the test for equality of specificities was 0.00251. The pooled AUC for blood specimen type was found to be 0.825.

**FIGURE 4:**
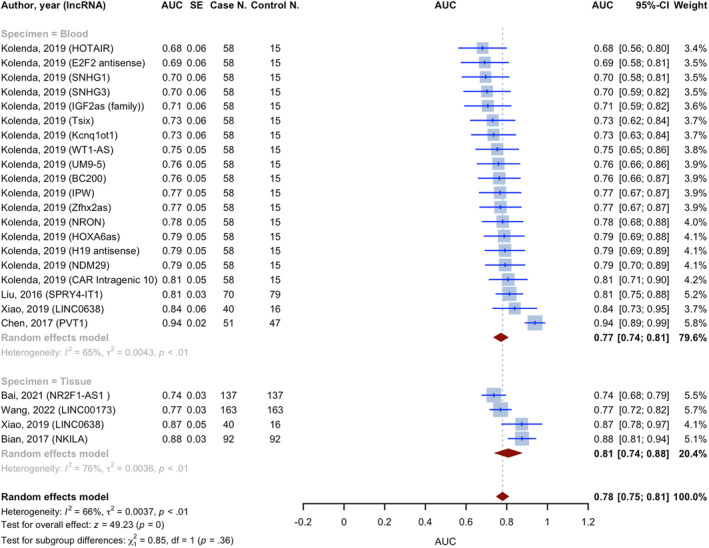
Forest plot of pooled AUCs, showing blood specimen and tissue specimen subgroups and combined diagnostic values.

All included studies in the analysis reported the AUC values for lncRNAs on the diagnosis of melanoma. The pooled AUC value was 0.780 (95% CI: 0.749–0.811, *p* < 0.0001, *I* ^2^ = 65.6%), calculated using the inverse variance method. This value was derived from 24 diagnostic accuracy evaluations and 23 individual lncRNAs, involving 1579 cases and 805 controls (Figure 4). The studies were classified into subgroups based on the specimen type used to measure lncRNA expression. The AUC for the blood specimen subgroup (*n* = 20), involving 1147 cases and 397 controls, was 0.772 (95% CI: 0.735–0.808; *I* ^2^ = 64.9%). For the tissue specimen subgroup (*n* = 4), involving 432 cases and 408 controls, the pooled AUC was 0.808 (95% CI: 0.740–0.876; *I* ^2^ = 76.3%). The test for between subgroup differences was not statistically significant (*p* = 0.3563) (Table 3).

**TABLE 3:** Summary of findings in the meta‐analysis.

Diagnosis										Prognosis					
Meta‐analysis	Specimen	No. of evaluations	Sensitivity [95% CI]	*p* Value	Specificity [95% CI]	*p*‐Value	AUC [95% CI]	*p*‐Value	*I* ^2^	Meta‐analysis	Specimen	No. of evaluations	HR [95% CI]	*I* ^2^	*p*‐Value
All lncRNAs															
Bivariant	Blood	19	0.720 [0.648; 0.783]	<0.001	0.794 [0.724; 0.851]	<0.001	0.825		18.7–33%	OS (HR >1)	Blood	9	2.606 [1.863; 3.645]	0.00%	
	Total	21	0.724 [0.659; 0.781]	<0.001	0.812 [0.752; 0.859]	<0.001	0.837		14.9–32.5%		Tissue	8	2.778 [2.218; 3.480]	0.00%	
											Total	17	2.723 [2.259; 3.283]	0.00%	< 0.0001
AUC	Blood	20					0.772 [0.735; 0.808]		64.90%	OS (HR <1)	Blood	2	0.348 [0.200; 0.607]	0.00%	0.0002
	Tissue	6					0.808 [0.740; 0.876]		76.30%	PFS (HR >1)	Blood	6	2.913 [2.050; 4.138]	0.00%	< 0.0001
	Total	24					0.780 [0.749; 0.811]	0	65.60%	PFS (HR <1)	Blood	4	0.457 [0.256; 0.817]	0.00%	0.0083
										DFS (HR >1)	Blood	4	2.760 [2.009; 3.792]	0.00%	< 0.0001

### 3.4 Meta‐analysis of the prognostic value of lncRNAs in melanoma patients

**FIGURE 5:**
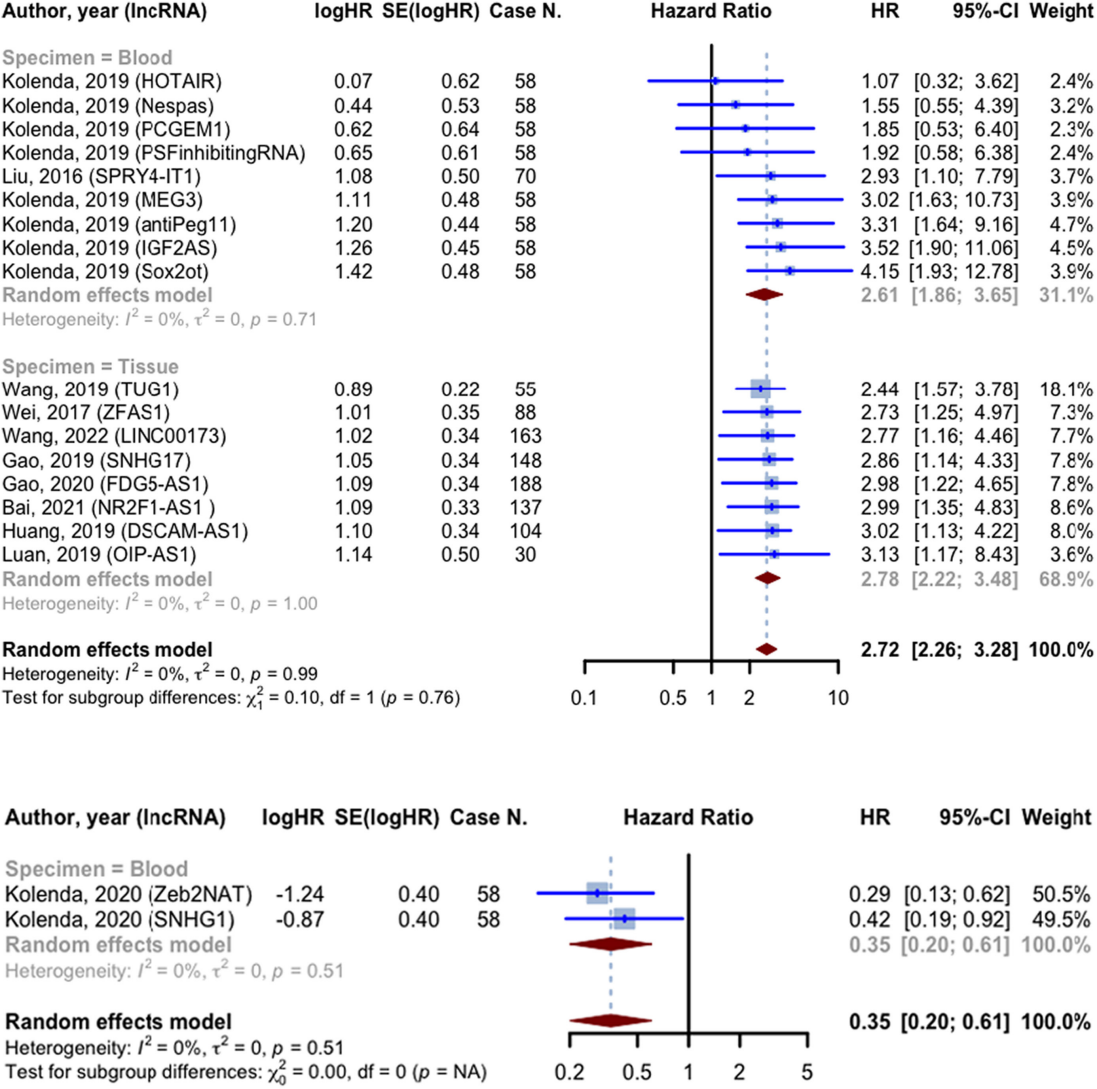
Forest plot of overall survival hazard ratios; prognostic evaluations were divided into two groups based on HR (less than or greater than one) and subgroups based on the specimen type.

Out of the 19 prognostic evaluations that reported OS hazard ratios, 17 reported HRs greater than 1. The combined HR for these studies was 2.723 (95% CI: 2.259–3.283, *p* < 0.0001; *I* ^2^ = 0.0%). The blood specimen subgroup (*n* = 9), involving 534 cases, had a pooled HR of 2.606 (95% CI: 1.863–3.645; *I* ^2^ = 0.0%). The tissue specimen subgroup (*n* = 8), involving 913 cases, had an HR of 2.778 (95% CI: 2.218–3.480; *I* ^2^ = 0.0%). The test for between subgroup differences was not statistically significant (*p* = 0.7571). The two studies reported HR smaller than 1 had pooled HR of 0.348 (95% CI: 0.200–0.607, *p* = 0.0002), involving 116 cases. Both studies utilized blood samples as specimens (Figure 5).

**FIGURE 6:**
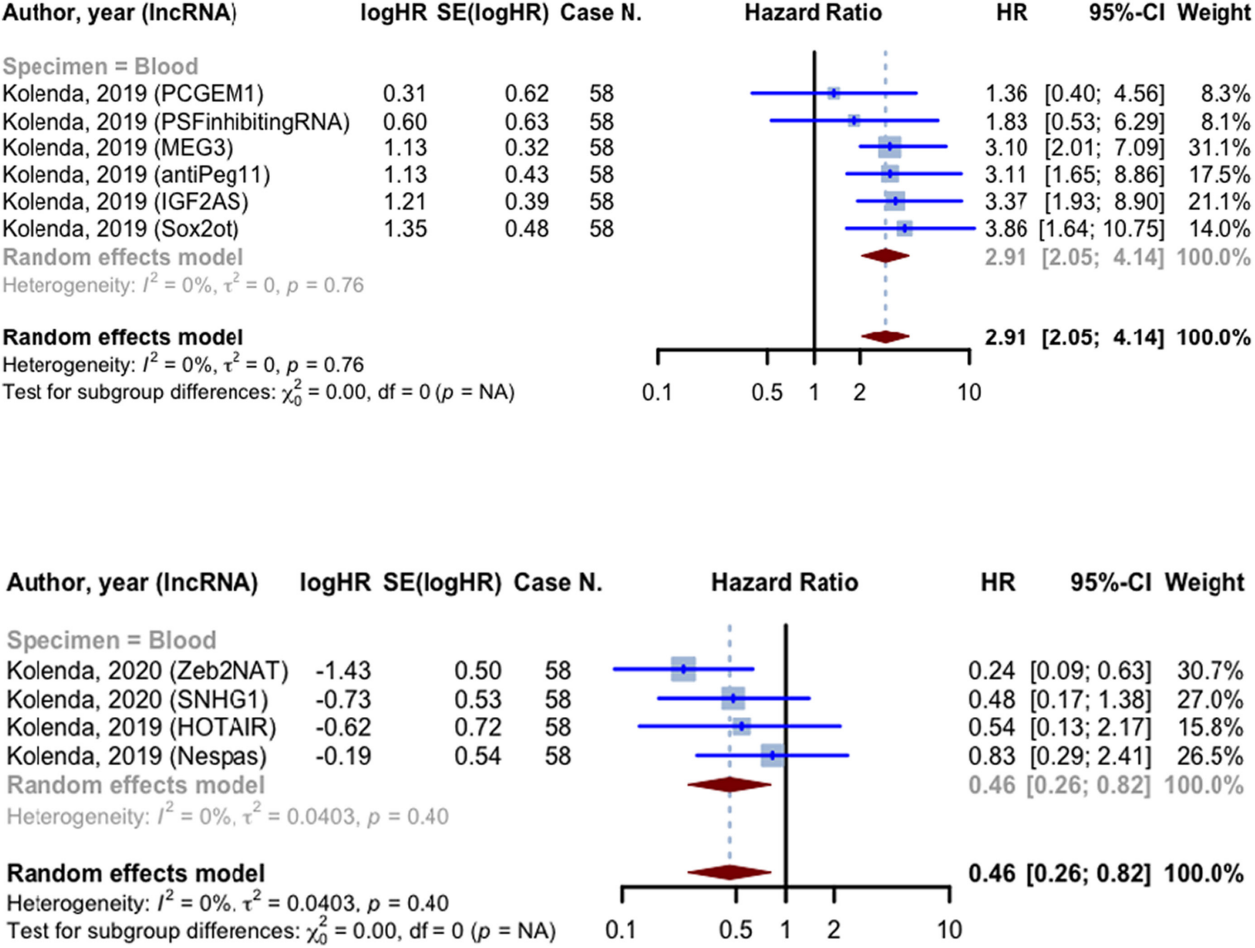
Forest plot of progression‐free survival hazard ratios; prognostic evaluations were divided into two groups based on HR (less than or greater than one) and subgroups based on specimen type.

Among the 10 prognostic evaluations that reported HRs for PFS, six indicated HR greater than 1 and involved 348 cases, all of which utilized blood samples as specimens. The pooled HR for the included studies was 2.913 (95% CI: 2.050–4.138, *p* < 0.0001; *I* ^2^ = 0.0%). The four studies that reported an HR smaller than 1, involving 232 cases, had a pooled HR of 0.457 (95% CI: 0.256–0.817, *p* = 0.0083). These studies employed blood samples as specimens (Figure 6).

**FIGURE 7:**
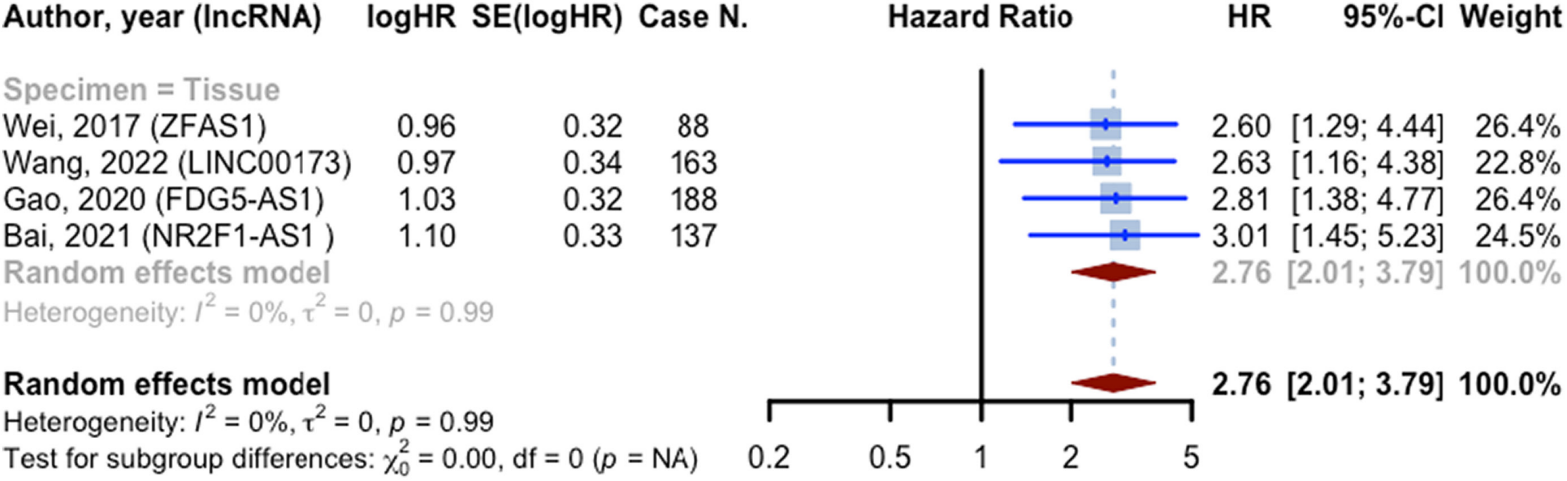
Forest plot of disease‐free survival hazard ratios; prognostic evaluations were divided into two groups based on HR (less than or greater than one) and subgroups based on specimen type.

All four prognostic evaluations that assessed DFS hazard ratios reported HRs greater than 1. These studies included 576 melanoma cases. Additionally, all of these evaluations utilized tissue samples as specimens. The pooled HR for the included studies was 2.760 (95% CI: 2.009–3.792, *p* < 0.0001; *I* ^2^ = 0.0%) (Figure 7) (Table 3).

## 4 DISCUSSION

Recently, accumulating evidence has shown that lncRNAs play a key role in various biological processes in different malignancies, including melanoma. Evidence has demonstrated that lncRNAs can be used as a biomarker for the early diagnosis, prognosis and treatment of melanoma, based on their role.^15^, ^16^, ^17^, ^18^ In this systematic review and meta‐analysis, we aimed to summarize the results of individual studies on human samples and investigate the diagnostic and prognostic value of lncRNAs in melanoma. Our meta‐analysis showed a cumulative sensitivity of 0.724, a pooled specificity of 0.812 and an overall AUC of 0.837 for lncRNAs in diagnosing melanoma. Regarding the type of specimen, there was no significant difference in the AUC of lncRNAs derived from tissue samples and those from serum. In the prognostic section, the combined HR for OS, PFS and DFS was 2.723 (95% CI: 2.259–3.283), 2.913 (95% CI: 2.050–4.138) and 2.760 (95% CI: 2.009–3.792), respectively. In our subgroup analysis, there was no significant difference in HR between tissue and blood samples.

Studies have shown that lncRNAs are involved in different cellular functions.^19^ Detection of large numbers of lncRNAs, their expression patterns in various types of malignancies, and their specificity and stability in body fluids suggest their potential role in developing novel diagnostic, prognostic and therapeutic tools for cancer.^20^ Currently, biopsy is the gold standard method of melanoma diagnosis.^21^, ^22^ It has been known that lncRNAs are secreted in body fluids. Despite histopathological biopsy, which is an invasive and uncomfortable method, lncRNAs can be easily obtained from patients. Thus, analysis of lncRNAs can be used as suitable diagnostic biomarkers for melanoma.

Multiple studies on melanoma cell lines have studied the mechanism of lncRNAs in melanoma. For example, in the study of Bian et al., NKILA, which was downregulated in melanoma tissue, suppressed the progression of the cell cycle and proliferation. Further, NKILA significantly induced apoptosis and inhibited invasion in melanoma cell lines through regulation of the nuclear factor kappa B (NF‐ĸB) signalling pathway.^9^ HOTAIR downregulation has been associated with inhibiting cellular proliferation and inducing apoptosis in melanoma cells through the regulation of NF‐ĸB.^23^ HOTAIR leads to melanoma cell growth and metastasis by sponging miR‐152‐3p and activating the PI3k/Akt/mTOR signalling pathway.^24^ Through investigation of the lncRNA PVT1 mechanism in uveal melanoma (UM) cell lines, it was revealed that the clonogenic capacity of cells significantly decreased after silencing lncRNA PVT1. Furthermore, it was demonstrated that PVT1 knockdown represses the proliferation and increases the apoptosis of UM cells through the downregulation of EZH2 expression.^25^ A study by Zhang et al. using the data from TCGA and GEO databases has suggested PRRT3‐AS1 as a potential diagnostic and prognostic biomarker for melanoma.^26^

Based on our results, lncRNAs could be used as a prognostic biomarker in melanoma patients. Similar to our results, studies on cell lines have shown that altered expression of TUG1,^27^ HOTAIR^28^, ^29^ and BANCR^30^ was related to malignant melanoma progression. Also, different studies on TCGA and GEO databases have demonstrated that additional lncRNAs, including PRRT3‐AS1,^26^ DANCR^26^ and FOXD2‐AS1,^31^ are related to clinical outcomes and melanoma progression. Additionally, several studies have introduced lncRNA‐based models for predicting clinical outcomes in uveal and cutaneous melanoma patients.^26^, ^32^, ^33^, ^34^, ^35^ Xu et al. created a novel signature based on five lncRNAs, including AATBC, AC145423.2, LINC01871, AC125807.2 and AC245041.1, that can predict the clinical outcome in melanoma patients.^34^ Also, Ma et al. reported a five ferroptosis‐related lncRNA signature with an AUC of 0.904 in a training cohort, which could be used as a potential prognostic biomarker for UM patients.^35^ Zhong et al. identified a model based on the 12 pyroptosis‐related lncRNA signature with the ability to predict the prognosis of cutaneous melanoma effectively. Thus, from these investigations, it could be concluded that using combinations of different lncRNAs with each other or other biomarkers could help clinicians make a more precise prediction of melanoma patients' prognosis.

To the best of our knowledge, our study is the first systematic review and meta‐analysis on the prognostic and diagnostic value of lncRNAs in melanoma. However, several limitations in this study should be mentioned. Firstly, only a few lncRNAs appeared in more than one study; thus, we were unable to conduct a meta‐analysis on the diagnostic and prognostic values of one type of lncRNA. Secondly, differences in methods, including different sample types, outcome measures, sample sizes and follow‐up periods in the studies, induced heterogeneity. The cut‐off value varied between studies, and although RT‐qPCR was used as the standard method to measure the expression level of lncRNAs, this may have caused heterogeneity in the results. Thirdly, we included the studies that obtained their samples directly from human subjects; studies that used data from databases were excluded from this study. Additionally, because most of the selected literature came from China, the results need further verification in other ethnicities.

Although physical examination and biopsy are considered the most reliable methods for diagnosing melanoma, the difficulties in differentiating between a benign mole and a melanoma underscore the necessity for supplementary tests to assist in the diagnostic process.^4^, ^36^, ^37^, ^38^ The timely identification and proactive measures of sun protection play a crucial role in mitigating the adverse health outcomes and fatalities linked to melanoma. Hence, the identification of disease‐associated biomarkers holds significant therapeutic and prognostic implications, particularly in the context of advanced‐stage melanoma. Early detection and intervention in this form of cancer can greatly enhance the chances of survival.^39^, ^40^, ^41^, ^42^ The findings presented in this study suggest that lncRNAs represent a newly recognized class of regulatory molecules that may have the potential to influence various aspects of melanoma, including proliferation, invasion, migration and apoptosis. Moreover, these molecules might play a direct role in the development of melanoma and contribute to the acquisition of drug resistance. As a result, lncRNAs hold promise as diagnostic and prognostic biomarkers for melanoma, and they may also serve as potential therapeutic targets in the future.

## 5 CONCLUSION

In conclusion, the current evidence shows that expression levels of some lncRNAs may vary in melanoma patients. Additionally, upregulation or downregulation of various lncRNAs is related to a patient's survival and melanoma prognosis. These findings suggest that lncRNAs should be considered as novel diagnostic and prognostic biomarkers for better management of patients in the future. However, more investigations are required to determine the prognostic and diagnostic value of lncRNAs for clinical use.

## AUTHOR CONTRIBUTIONS

**Mahdi Masrour:** Conceptualization (equal); data curation (equal); formal analysis (equal); investigation (equal); methodology (equal); project administration (equal); resources (equal); software (equal); supervision (equal); validation (equal); visualization (equal); writing – original draft (equal); writing – review and editing (equal). **Shaghayegh Khanmohammadi:** Conceptualization (equal); data curation (equal); formal analysis (equal); investigation (equal); methodology (equal); project administration (equal); resources (equal); software (equal); supervision (equal); validation (equal); visualization (equal); writing – original draft (equal); writing – review and editing (equal). **Parisa Fallahtafti:** Data curation (equal); investigation (equal); project administration (equal); validation (equal); visualization (equal); writing – original draft (equal); writing – review and editing (equal). **Seyedeh Melika Hashemi:** Data curation (equal); investigation (equal); project administration (equal); validation (equal); visualization (equal); writing – original draft (equal); writing – review and editing (equal). **Nima Rezaei:** Conceptualization (equal); methodology (equal); project administration (equal); resources (equal); supervision (equal); validation (equal); writing – original draft (equal); writing – review and editing (equal).

## FUNDING INFORMATION

This research received no specific grant from any funding agency in the public, commercial, or not‐for‐profit sectors.

## CONFLICT OF INTEREST STATEMENT

The authors declare that they have no competing interests.

## Supporting information


Tables S1–S2

## Data Availability

All relevant data are within the paper and its Supporting Information files.
