# Supplementary material for: Long non‐coding RNA as a potential diagnostic and prognostic biomarker in melanoma: A systematic review and meta‐analysis
Source: J Cell Mol Med. 2024 Jan 9;28(3):e18109. doi: 10.1111/jcmm.18109 (PMC10844705; doi:10.1111/jcmm.18109)
Supplement: Supplementary file 1 — Tables S1–S2 [file JCMM-28-e18109-s001.docx]

**Supplementary Table 1.** Search strategy for each database

| **Query** |
| --- |
| **PubMed** |
| (“Melanoma”[MeSH] OR “Melanoma”[tiab] OR “Melanomas”[tiab] OR “Malignant Melanoma”[tiab] OR “Malignant Melanomas”[tiab] OR “Melanoma, Malignant”[tiab] OR “Melanomas, Malignant”[tiab])  AND  (“Noncoding RNA, Long”[tiab] OR “lncRNA”[tiab] OR “Long ncRNA”[tiab] OR “ncRNA, Long”[tiab] OR “RNA, Long Non-Translated”[tiab] OR “Long Non-Translated RNA”[tiab] OR “Non-Translated RNA, Long”[tiab] OR “RNA, Long Non Translated”[tiab] OR “Long Non-Coding RNA”[tiab] OR “Long Non Coding RNA”[tiab] OR “Non-Coding RNA, Long”[tiab] OR “RNA, Long Non-Coding”[tiab] OR “Long Non-Protein-Coding RNA”[tiab] OR “Long Non Protein Coding RNA”[tiab] OR “Non-Protein-Coding RNA, Long”[tiab] OR “RNA, Long Non-Protein-Coding”[tiab] OR “Long Noncoding RNA”[tiab] OR “RNA, Long Untranslated”[tiab] OR “Long Untranslated RNA”[tiab] OR “Untranslated RNA, Long”[tiab] OR “Long ncRNAs”[tiab] OR “ncRNAs, Long”[tiab] OR “Long Intergenic Non-Protein Coding RNA”[tiab] OR “Long Intergenic Non Protein Coding RNA”[tiab] OR “LincRNAs”[tiab] OR “LINC RNA”[tiab] OR “LincRNA”[tiab] OR “RNA, Long Noncoding”[tiab] OR RNA, Long Noncoding[MeSH]) |
| **ISI** |
| TS=(“Noncoding RNA, Long” OR “lncRNA” OR “Long ncRNA” OR “ncRNA, Long” OR “RNA, Long Non-Translated” OR “Long Non-Translated RNA” OR “Non-Translated RNA, Long” OR “RNA, Long Non Translated” OR “Long Non-Coding RNA” OR “Long Non Coding RNA” OR “Non-Coding RNA, Long” OR “RNA, Long Non-Coding” OR “Long Non-Protein-Coding RNA” OR “Long Non Protein Coding RNA” OR “Non-Protein-Coding RNA, Long” OR “RNA, Long Non-Protein-Coding” OR “Long Noncoding RNA” OR “RNA, Long Untranslated” OR “Long Untranslated RNA” OR “Untranslated RNA, Long” OR “Long ncRNAs” OR “ncRNAs, Long” OR “Long Intergenic Non-Protein Coding RNA” OR “Long Intergenic Non Protein Coding RNA” OR “LincRNAs” OR “LINC RNA” OR “LincRNA”)  AND  TS=(“Melanoma” OR “Melanomas” OR “Malignant Melanoma” OR “Malignant Melanomas” OR “Melanoma, Malignant” OR “Melanomas, Malignant”) |
| **Scopus** |
| TITLE-ABS-KEY(“Noncoding RNA, Long” OR “lncRNA” OR “Long ncRNA” OR “ncRNA, Long” OR “RNA, Long Non-Translated” OR “Long Non-Translated RNA” OR “Non-Translated RNA, Long” OR “RNA, Long Non Translated” OR “Long Non-Coding RNA” OR “Long Non Coding RNA” OR “Non-Coding RNA, Long” OR “RNA, Long Non-Coding” OR “Long Non-Protein-Coding RNA” OR “Long Non Protein Coding RNA” OR “Non-Protein-Coding RNA, Long” OR “RNA, Long Non-Protein-Coding” OR “Long Noncoding RNA” OR “RNA, Long Untranslated” OR “Long Untranslated RNA” OR “Untranslated RNA, Long” OR “Long ncRNAs” OR “ncRNAs, Long” OR “Long Intergenic Non-Protein Coding RNA” OR “Long Intergenic Non Protein Coding RNA” OR “LincRNAs” OR “LINC RNA” OR “LincRNA”)  AND  TITLE-ABS-KEY(“Melanoma” OR “Melanomas” OR “Malignant Melanoma” OR “Malignant Melanomas” OR “Melanoma, Malignant” OR “Melanomas, Malignant”) |
| **EMBASE** |
| (“Noncoding RNA, Long”:ti,ab,kw OR “lncRNA”:ti,ab,kw OR “Long ncRNA”:ti,ab,kw OR “ncRNA, Long”:ti,ab,kw OR “RNA, Long Non-Translated”:ti,ab,kw OR “Long Non-Translated RNA”:ti,ab,kw OR “Non-Translated RNA, Long”:ti,ab,kw OR “RNA, Long Non Translated”:ti,ab,kw OR “Long Non-Coding RNA”:ti,ab,kw OR “Long Non Coding RNA”:ti,ab,kw OR “Non-Coding RNA, Long”:ti,ab,kw OR “RNA, Long Non-Coding”:ti,ab,kw OR “Long Non-Protein-Coding RNA”:ti,ab,kw OR “Long Non Protein Coding RNA”:ti,ab,kw OR “Non-Protein-Coding RNA, Long”:ti,ab,kw OR “RNA, Long Non-Protein-Coding”:ti,ab,kw OR “Long Noncoding RNA”:ti,ab,kw OR “RNA, Long Untranslated”:ti,ab,kw OR “Long Untranslated RNA”:ti,ab,kw OR “Untranslated RNA, Long”:ti,ab,kw OR “Long ncRNAs”:ti,ab,kw OR “ncRNAs, Long”:ti,ab,kw OR “Long Intergenic Non-Protein Coding RNA”:ti,ab,kw OR “Long Intergenic Non Protein Coding RNA”:ti,ab,kw OR “LincRNAs”:ti,ab,kw OR “LINC RNA”:ti,ab,kw OR “LincRNA”:ti,ab,kw)  AND  (“Melanoma”:ti,ab,kw OR “Melanomas”:ti,ab,kw OR “Malignant Melanoma”:ti,ab,kw OR “Malignant Melanomas”:ti,ab,kw OR “Melanoma, Malignant”:ti,ab,kw OR “Melanomas, Malignant”:ti,ab,kw) |

**Supplementary Table 2.** Excluded Studies

| **ID** | **Author, year** | **Title** | **Reason of exclusion** |
| --- | --- | --- | --- |
|  | An, 2022 (1) | lncRNA AGAP2-AS1 Facilitates Tumorigenesis and Ferroptosis Resistance through SLC7A11 by IGF2BP2 Pathway in Melanoma | Data from dataset |
|  | B. J. Wang, 2018 (2) | Long noncoding RNA PVT1 promotes melanoma progression via endogenous sponging miR-26b | Data was reported as charts and the numbers were not given. |
|  | Bi, 2020 (3) | SNHG16 promotes cell proliferation and migration through sponging MIR-132 in melanoma | Full text unavailable |
|  | Botti, 2016 (4) | The long noncoding RNA HOTAIR is associated to metastatic progression of melanoma and it can be identified in the blood of patients with advanced disease | Data was reported as charts and the numbers were not given. |
|  | Chen L, 2018 (5) | LncRNA GAS5 regulates redox balance and dysregulates the cell cycle and apoptosis in malignant melanoma cells | No related survival/diagnostic performance analysis |
|  | Chen X, 2017 (6) | Long noncoding RNA ILF3-AS1 promotes cell proliferation, migration, and invasion via negatively regulating miR-200b/a/429 in melanoma | No related survival/diagnostic performance analysis |
|  | Chen X, 2017 (7) | Long noncoding RNA MHENCR promotes melanoma progression via regulating miR-425/489-mediated PI3K-Akt pathway | No related survival/diagnostic performance analysis |
|  | Chen X, 2019 (8) | LncRNA FOXD3-AS1 promotes proliferation, invasion and migration of cutaneous malignant melanoma via regulating miR-325/MAP3K2 | No related survival/diagnostic performance analysis |
|  | Chen X, 2020 (9) | Regulation of melanoma malignancy by the RP11-705C15.3/miR-145-5p/NRAS/MAPK signaling axis | No related survival/diagnostic performance analysis |
|  | Chen X, 2021 (10) | DUXAP8 knockdown inhibits the development of melanoma by regulating the miR-3182/NUPR1 pathway | Data was reported as charts and the numbers were not given. |
|  | Coe, 2019 (11) | The MITF-SOX10 regulated long non-coding RNA DIRC3 is a melanoma tumour suppressor | Data from dataset |
|  | G. Shi, 2018 (12) | lncRNA H19 predicts poor prognosis in patients with melanoma and regulates cell growth, invasion, migration and epithelial-mesenchymal transition in melanoma cells | Data was reported as charts and the numbers were not given. |
|  | Gao, 2018 (13) | The positive feedback loop between ILF3 and lncRNA ILF3-AS1 promotes melanoma proliferation, migration, and invasion | No related survival/diagnostic performance analysis |
|  | H. L. Xu, 2020 (14) | Clinical significance of lncRNA MIR31HG in melanoma | Data was reported as charts and the numbers were not given. |
|  | H. Yang (15) | ZNF667‐AS1, a positively regulating MEGF10, inhibits the progression of uveal melanoma by modulating cellular aggressiveness | Data from dataset |
|  | H. Zhang (16) | LncRNA HOXD-AS1 promotes melanoma cell proliferation and invasion by suppressing RUNX3 expression | Data was reported as charts and the numbers were not given. |
|  | H. Zhao (17) | Long noncoding RNA HEIH promotes melanoma cell proliferation, migration and invasion via inhibition of miR-200b/a/429 | Data was reported as charts and the numbers were not given. |
|  | Huo J, 2022 (18) | The LncRNA MIR155HG is Upregulated by SP1 in Melanoma Cells and Drives Melanoma Progression via Modulating the MiR-485-3p/PSIP1 Axis | Full text unavailable |
|  | Ichigozaki, 2016 (19) | Serum long non-coding RNA, snoRNA host gene 5 level as a new tumor marker of malignant melanoma | Letter to the editorial |
|  | J. Oliver, 2022 (20) | Association of Circular RNA and Long Non-Coding RNA Dysregulation with the Clinical Response to Immune Checkpoint Blockade in Cutaneous Metastatic Melanoma | No related data |
|  | J. Xie, 2020 (21) | Long Noncoding RNA CAR10 Contributes to Melanoma Progression By Suppressing miR-125b-to Induce RAB3D | Data was reported as charts and the numbers were not given. |
|  | J. Xu, 2022 (22) | LINC00518 affects the proliferation, invasion and migration of cutaneous malignant melanoma cells via miR-526b-3p/EIF5A2 axis | Data was reported as charts and the numbers were not given. |
|  | J. Xu, 2022 (23) | LINC01063 functions as an oncogene in melanoma through regulation of miR-5194-mediated SOX12 expression | No related survival/diagnostic performance analysis |
|  | J. Y. Zhu (24) | Reconstruction of lncRNA-miRNA-mRNA network based on competitive endogenous RNA reveals functional lncRNAs in skin cutaneous melanoma | Data from dataset |
|  | Jiao, 2018 (25) | Upregulation of LINC00963 facilitates melanoma progression through miR-608/NACC1 pathway and predicts poor prognosis | Data was reported as charts and the numbers were not given. |
|  | K. Schmidt, 2016 (26) | The lncRNA SLNCR1 Mediates Melanoma Invasion through a Conserved SRA1-like Region | Data from dataset |
|  | L. Song, 2021 (27) | Screening and survival analysis of melanoma immunodrug response-related genes and the function of magnetic nanoparticles in gene extraction | Data from dataset |
|  | L. Wen, 2019 (28) | Increased expression of long noncoding RNA GAS6-AS2 promotes proliferation and inhibits apoptosis of melanoma cells via upregulating GAS6 expression | Data was reported as charts and the numbers were not given. |
|  | L. Wu, 2020 (29) | LncRNA MEG3 promotes melanoma growth, metastasis and formation through modulating miR-21/E-cadherin axis | Data was reported as charts and the numbers were not given. |
|  | Li X, 2018 (30) | Long non-coding RNA PANDAR promotes melanoma cell invasion through regulating epithelial-mesenchymal transition | No related survival/diagnostic performance analysis |
|  | Li, 2014 (31) | Long non-coding RNA BANCR promotes proliferation in malignant melanoma by regulating MAPK pathway activation | Duplicate data with another included study |
|  | Liang, 2019 (32) | Long noncoding RNA ZFAS1 promotes tumorigenesis through regulation of miR-150-5p/RAB9A in melanoma | No related survival/diagnostic performance analysis |
|  | Liu Y, 2021 (33) | Long noncoding RNA LINC00518 induces radioresistance by regulating glycolysis through an miR-33a-3p/HIF-1α negative feedback loop in melanoma | Data from dataset |
|  | Long, 2018 (34) | lncRNA-MEG3 Suppresses the Proliferation and Invasion of Melanoma by Regulating CYLD Expression Mediated by Sponging miR-499-5p | No related survival/diagnostic performance analysis |
|  | Lu W, 2018 (35) | INC00888 promoted tumorigenicity of melanoma via miR-126/CRK signaling axis | No related survival/diagnostic performance analysis |
|  | Luan W, 2018 (36) | Long non-coding RNA H19 promotes glucose metabolism and cell growth in malignant melanoma via miR-106a-5p/E2F3 axis | No related survival/diagnostic performance analysis |
|  | Mou, 2019 (37) | LNMAT1 Promotes Invasion-Metastasis Cascade in Malignant Melanoma by Epigenetically Suppressing CADM1 Expression | No related survival/diagnostic performance analysis |
|  | N. Ni, 2017 (38) | Up-regulation of long noncoding RNA FALEC predicts poor prognosis and promotes melanoma cell proliferation through epigenetically silencing p21 | Data was reported as charts and the numbers were not given. |
|  | P. Wang, 2020 (39) | Comprehensive Analysis of the Tumor Microenvironment in Cutaneous Melanoma associated with Immune Infiltration | Data from dataset |
|  | P. Wang, 2020 (40) | LncRNA MALAT1 promotes the proliferation, migration, and invasion of melanoma cells by downregulating miR-23a | Data was reported as charts and the numbers were not given. |
|  | Q. Peng, 2020 (41) | A LHFPL3-AS1/miR-580-3p/STAT3 feedback loop promotes the malignancy in melanoma via activation of JAK2/STAT3 signaling | Data was reported as charts and the numbers were not given. |
|  | Q. Wang, 2019 (42) | Long non-coding RNA ZEB1-AS1 indicates poor prognosis and promotes melanoma progression through targeting miR-1224-5p | Data was reported as charts and the numbers were not given. |
|  | W. Xu, 2020 (43) | Long non-coding RNA GAS5 accelerates oxidative stress in melanoma cells by rescuing EZH2-mediated CDKN1C downregulation | Data was reported as charts and the numbers were not given. |
|  | X. Q. Xia, 2020 (44) | LINC00662 promotes cell proliferation, migration and invasion of melanoma by sponging miR-890 to upregulate ELK3 | Data from dataset |
|  | X. Wei, 2019 (45) | Long noncoding RNA HCP5 suppresses skin cutaneous melanoma development by regulating rarres3 gene expression via sponging miR-12 | Data was reported as charts and the numbers were not given. |
|  | X. Z. Xu (46) | SMG7-AS1 as a prognostic biomarker and predictor of immunotherapy responses for skin cutaneous melanoma | Data from dataset |
|  | X. Zhou 2019 (47) | Long noncoding RNA CPS1‐IT1 suppresses melanoma cell metastasis through inhibiting Cyr61 via competitively binding to BRG1 | Data was reported as charts and the numbers were not given. |
|  | Y. Wang, 2020 (48) | Long noncoding RNA TTN-AS1 facilitates tumorigenesis and metastasis by maintaining TTN expression in skin cutaneous melanoma | Data from dataset |
|  | Y. Wang, 2021 (49) | STAT3-induced ZBED3-AS1 promotes the malignant phenotypes of melanoma cells by activating PI3K/AKT signaling pathway | Data from dataset |

1. An LF, Huang JW, Ge SH, Zhang X, Wang J. lncRNA AGAP2-AS1 Facilitates Tumorigenesis and Ferroptosis Resistance through SLC7A11 by IGF2BP2 Pathway in Melanoma. COMPUTATIONAL AND MATHEMATICAL METHODS IN MEDICINE. 2022;2022.

2. Wang BJ, Ding HW, Ma GA. Long noncoding RNA PVT1 promotes melanoma progression via endogenous sponging miR-26b. Oncology Research. 2018;26(5):675-81.

3. Bi LL, Hua XQ, Li WH, Wang L, Li Y, Jia XF. SNHG16 promotes cell proliferation and migration through sponging MIR-132 in melanoma. Journal of Biological Regulators and Homeostatic Agents. 2020;34(4):1307-16.

4. Botti C, Scognamiglio G, Marra L, Aquino G, Falcone R, Anniciello A, et al. The long noncoding RNA HOTAIR is associated to metastatic progression of melanoma and it can be identified in the blood of patients with advanced disease. JOURNAL OF TRANSLATIONAL MEDICINE. 2016;14.

5. Chen LM, Ma DM, Li YY, Li XY, Zhao L, Zhang J, et al. Effect of long non-coding RNA PVT1 on cell proliferation and migration in melanoma. INTERNATIONAL JOURNAL OF MOLECULAR MEDICINE. 2018;41(3):1275-82.

6. Chen X, Liu S, Zhao X, Ma X, Gao G, Yu L, et al. Long noncoding RNA ILF3-AS1 promotes cell proliferation, migration, and invasion via negatively regulating miR-200b/a/429 in melanoma. Bioscience Reports. 2017;37(6).

7. Chen XJ, Dong H, Liu S, Yu L, Yan DX, Yao XW, et al. Long noncoding RNA MHENCR promotes melanoma progression via regulating miR-425/489-mediated PI3K-Akt pathway. AMERICAN JOURNAL OF TRANSLATIONAL RESEARCH. 2017;9(1):90-102.

8. Chen XG, Gao J, Yu YG, Zhao ZJ, Pan YL. LncRNA FOXD3-AS1 promotes proliferation, invasion and migration of cutaneous malignant melanoma via regulating miR-325/MAP3K2. BIOMEDICINE & PHARMACOTHERAPY. 2019;120.

9. Chen XE, Chen P, Chen SS, Lu J, Ma T, Shi G, et al. Long non-coding RNA FENDRR inhibits migration and invasion of cutaneous malignant melanoma cells. BIOSCIENCE REPORTS. 2020;40.

10. Chen XJ, Liu S, Han DM, Han DZ, Sun WJ, Zhao XC. Regulation of melanoma malignancy by the RP11-705C15.3/miR-145-5p/NRAS/MAPK signaling axis. Cancer Gene Therapy. 2021;28(10-11):1198-212.

11. Coe EA, Tan JY, Shapiro M, Louphrasitthiphol P, Bassett AR, Marques AC, et al. The MITF-SOX10 regulated long non-coding RNA DIRC3 is a melanoma tumour suppressor. PLoS Genetics. 2019;15(12).

12. Shi G, Li H, Gao F, Tan Q. lncRNA H19 predicts poor prognosis in patients with melanoma and regulates cell growth, invasion, migration and epithelial-mesenchymal transition in melanoma cells. Onco Targets Ther. 2018;11:3583-95.

13. Gao YN, Yu H, Liu YH, Liu XB, Zheng J, Ma J, et al. Long Non-Coding RNA HOXA-AS2 Regulates Malignant Glioma Behaviors and Vasculogenic Mimicry Formation via the MiR-373/EGFR Axis. CELLULAR PHYSIOLOGY AND BIOCHEMISTRY. 2018;45(1):131-47.

14. Xu HL, Tian FZ. Clinical significance of lncRNA MIR31HG in melanoma. European Review for Medical and Pharmacological Sciences. 2020;24(8):4389-95.

15. Yang H, Cai MY, Rong H, Ma LR, Xu YL. ZNF667-AS1, a positively regulating MEGF10, inhibits the progression of uveal melanoma by modulating cellular aggressiveness. JOURNAL OF BIOCHEMICAL AND MOLECULAR TOXICOLOGY. 2021;35(5).

16. Zhang H, Bai M, Zeng A, Si L, Yu N, Wang X. LncRNA HOXD-AS1 promotes melanoma cell proliferation and invasion by suppressing RUNX3 expression. American Journal of Cancer Research. 2017;7(12):2526-35.

17. Zhao H, Xing G, Wang Y, Luo Z, Liu G, Meng H. Long noncoding RNA HEIH promotes melanoma cell proliferation, migration and invasion via inhibition of miR-200b/a/429. Bioscience Reports. 2017;37(3).

18. Huo J, Wang Y, Zhang YF, Wang W, Yang PW, Zhao WW, et al. The LncRNA MIR155HG is Upregulated by SP1 in Melanoma Cells and Drives Melanoma Progression via Modulating the MiR-485-3p/PSIP1 Axis. ANTI-CANCER AGENTS IN MEDICINAL CHEMISTRY. 2022;22(1):152-9.

19. Ichigozaki Y, Fukushima S, Jinnin M, Miyashita A, Nakahara S, Tokuzumi A, et al. Serum long non-coding RNA, snoRNA host gene 5 level as a new tumor marker of malignant melanoma. Experimental Dermatology. 2016;25(1):67-9.

20. Oliver J, Onieva JL, Garrido-Barros M, Berciano-Guerrero M, Sánchez-Muñoz A, José Lozano M, et al. Association of Circular RNA and Long Non-Coding RNA Dysregulation with the Clinical Response to Immune Checkpoint Blockade in Cutaneous Metastatic Melanoma. Biomedicines. 2022;10(10).

21. Xie J, Zheng YY, Xu XM, Sun CC, Lv MF. Long Noncoding RNA CAR10 Contributes to Melanoma Progression By Suppressing miR-125b-to Induce RAB3D. ONCOTARGETS AND THERAPY. 2020;13:6203-11.

22. Xu J, Zhang F, Lin M, Tong Y. LINC00518 affects the proliferation, invasion and migration of cutaneous malignant melanoma cells via miR-526b-3p/EIF5A2 axis. Acta Biochim Pol. 2022;69(1):101-11.

23. Xu J, Ou R, Nie G, Wen J, Ling L, Mo L, et al. LINC01063 functions as an oncogene in melanoma through regulation of miR-5194-mediated SOX12 expression. Melanoma Research. 2022;32(4):218-30.

24. Zhu JJ, Xu P. Long Noncoding RNA Small Nucleolar RNA Host Gene 6 Promotes Cell Proliferation, Migration and Invasion in Melanoma by Sponging miR-944. JOURNAL OF BIOMATERIALS AND TISSUE ENGINEERING. 2021;11(8):1459-65.

25. Jiao HL, Jiang SY, Wang H, Li YC, Zhang W. Upregulation of LINC00963 facilitates melanoma progression through miR-608/NACC1 pathway and predicts poor prognosis. BIOCHEMICAL AND BIOPHYSICAL RESEARCH COMMUNICATIONS. 2018;504(1):34-9.

26. Schmidt K, Joyce CE, Buquicchio F, Brown A, Ritz J, Distel RJ, et al. The lncRNA SLNCR1 Mediates Melanoma Invasion through a Conserved SRA1-like Region. Cell Reports. 2016;15(9):2025-37.

27. Song L, Du N, Luo H, Li F. Screening and survival analysis of melanoma immunodrug response-related genes and the function of magnetic nanoparticles in gene extraction. Materials Express. 2021;11(8):1306-12.

28. Wen L, Zheng Y, Wen X, Zhang Y, Zeng W. Increased expression of long noncoding RNA GAS6-AS2 promotes proliferation and inhibits apoptosis of melanoma cells via upregulating GAS6 expression. IUBMB Life. 2019;71(10):1503-14.

29. Wu LC, Zhu LF, Li YC, Zheng ZX, Lin X, Yang CY. LncRNA MEG3 promotes melanoma growth, metastasis and formation through modulating miR-21/E-cadherin axis (vol 20, 12, 2020). CANCER CELL INTERNATIONAL. 2020;20(1).

30. Li X, Zhang L, Song P, Xu J, Li G. Long non-coding RNA PANDAR promotes melanoma cell invasion through regulating epithelial-mesenchymal transition. Int J Clin Exp Pathol. 2018;11(5):2430-9.

31. Li R, Zhang L, Jia L, Duan Y, Li Y, Bao L, et al. Long non-coding RNA BANCR promotes proliferation in malignant melanoma by regulating MAPK pathway activation. PLoS ONE. 2014;9(6).

32. Liang L, Zhang Z, Qin X, Gao Y, Zhao P, Liu J, et al. Long noncoding RNA ZFAS1 promotes tumorigenesis through regulation of miR-150-5p/RAB9A in melanoma. Melanoma Research. 2019;29(6):569-81.

33. Liu Y, He D, Xiao M, Zhu Y, Zhou J, Cao K. Long noncoding RNA LINC00518 induces radioresistance by regulating glycolysis through an miR-33a-3p/HIF-1α negative feedback loop in melanoma. Cell Death and Disease. 2021;12(3).

34. Long JW, Menggen QQG, Wuren QMG, Shi Q, Pi XM. Long Noncoding RNA Taurine-Upregulated Gene1 (TUG1) Promotes Tumor Growth and Metastasis Through TUG1/Mir-129-5p/Astrocyte-Elevated Gene-1 (AEG-1) Axis in Malignant Melanoma. MEDICAL SCIENCE MONITOR. 2018;24:1547-59.

35. Lu W, Tao X, Fan Y, Tang Y, Xu X, Fan S, et al. LINC00888 promoted tumorigenicity of melanoma via miR-126/CRK signaling axis. OncoTargets and Therapy. 2018;11:4431-42.

36. Luan W, Zhou Z, Ni X, Xia Y, Wang J, Yan Y, et al. Long non-coding RNA H19 promotes glucose metabolism and cell growth in malignant melanoma via miR-106a-5p/E2F3 axis. Journal of Cancer Research and Clinical Oncology. 2018;144(3):531-42.

37. Mou K, Zhang X, Mu X, Ge R, Han D, Zhou Y, et al. LNMAT1 Promotes Invasion-Metastasis Cascade in Malignant Melanoma by Epigenetically Suppressing CADM1 Expression. Frontiers in Oncology. 2019;9.

38. Ni N, Song H, Wang X, Xu X, Jiang Y, Sun J. Up-regulation of long noncoding RNA FALEC predicts poor prognosis and promotes melanoma cell proliferation through epigenetically silencing p21. Biomedicine and Pharmacotherapy. 2017;96:1371-9.

39. Wang P, Hu L, Fu G, Lu J, Zheng Y, Li Y, et al. LncRNA MALAT1 promotes the proliferation, migration, and invasion of melanoma cells by downregulating miR-23a. Cancer Management and Research. 2020;12:6553-62.

40. Wang P, Zhang X, Sun N, Zhao Z, He J. Comprehensive analysis of the tumor microenvironment in cutaneous melanoma associated with immune infiltration. Journal of Cancer. 2020;11(13):3858-70.

41. Peng Q, Liu L, Pei H, Zhang J, Chen M, Zhai X. A LHFPL3-AS1/miR-580-3p/STAT3 feedback loop promotes the malignancy in melanoma via activation of JAK2/STAT3 signaling. Molecular Cancer Research. 2020;18(11):1724-34.

42. Wang Q, Zhang R, Liu D. Long non-coding RNA ZEB1-AS1 indicates poor prognosis and promotes melanoma progression through targeting miR-1224-5p. Experimental and Therapeutic Medicine. 2019;17(1):857-62.

43. Xu W, Yan Z, Hu F, Wei W, Yang C, Sun Z. Long non-coding RNA GAS5 accelerates oxidative stress in melanoma cells by rescuing EZH2-mediated CDKN1C downregulation. Cancer Cell International. 2020;20(1).

44. Xia XQ, Lu WL, Ye YY, Chen J. LINC00662 promotes cell proliferation, migration and invasion of melanoma by sponging miR-890 to upregulate ELK3. Eur Rev Med Pharmacol Sci. 2020;24(16):8429-38.

45. Wei X, Gu X, Ma M, Lou C. Long noncoding RNA HCP5 suppresses skin cutaneous melanoma development by regulating rarres3 gene expression via sponging miR-12. OncoTargets and Therapy. 2019;12:6323-35.

46. Xu XZ, Ju YK, Zhao XH, Yang P, Zhu F, Fang BR. SMG7-AS1 as a prognostic biomarker and predictor of immunotherapy responses for skin cutaneous melanoma. GENOMICS. 2023;115(3).

47. Zhou X, Rao Y, Sun Q, Liu Y, Chen J, Bu W. Long noncoding RNA CPS1-IT1 suppresses melanoma cell metastasis through inhibiting Cyr61 via competitively binding to BRG1. Journal of Cellular Physiology. 2019;234(12):22017-27.

48. Wang Y, Li D, Lu J, Chen L, Zhang S, Qi W, et al. Long noncoding RNA TTN-AS1 facilitates tumorigenesis and metastasis by maintaining TTN expression in skin cutaneous melanoma. Cell Death and Disease. 2020;11(8).

49. Wang Y, Ba HJ, Wen XZ, Zhou M, Küçük C, Tamagnone L, et al. A prognostic model for melanoma patients on the basis of immune-related lncRNAs. Aging. 2021;13(5):6554-64.
